# The Relation between Sustained Attention and Incidental and Intentional Object-Location Memory

**DOI:** 10.3390/brainsci10030145

**Published:** 2020-03-04

**Authors:** Efrat Barel, Orna Tzischinsky

**Affiliations:** Department of Behavioral Sciences, The Max Stern Academic College of Emek Yezreel, Emek Yezreel 19300, Israel; orna@yvc.ac.il

**Keywords:** object-location memory, sustained attention, incidental encoding, intentional encoding

## Abstract

The role of attention allocation in object-location memory has been widely studied through incidental and intentional encoding conditions. However, the relation between sustained attention and memory encoding processes has scarcely been studied. The present study aimed to investigate performance differences across incidental and intentional encoding conditions using a divided attention paradigm. Furthermore, the study aimed to examine the relation between sustained attention and incidental and intentional object-location memory performance. Based on previous findings, an all women sample was recruited in order to best illuminate the potential effects of interest. Forty-nine women participated in the study and completed the psychomotor vigilance test, as well as object-location memory tests, under both incidental and intentional encoding divided attention conditions. Performance was higher in the incidental encoding condition than in the intentional encoding condition. Furthermore, sustained attention correlated with incidental, but not with intentional memory performance. These findings are discussed in light of the automaticity hypothesis, specifically as it regards the role of attention allocation in encoding object-location memory. Furthermore, the role of sustained attention in incidental memory performance is discussed in light of previous animal and human studies that have examined the brain regions involved in these cognitive processes. We conclude that under conditions of increased mental demand, executive attention is associated with incidental, but not with intentional encoding, thus identifying the exact conditions under which executive attention influence memory performance.

## 1. Introduction

Object-location memory is a complex neurocognitive ability that presents a challenge for our cognitive system. It involves three components: (1) object-processing, (2) spatial-location processing, and (3) object-location binding [1]. Object-location memory is a fundamental ability that is needed in our daily lives. Given its adaptive value for both humans and animals, it has been suggested that object-location memory is not only driven by conscious recollections of objects’ locations, but rather that it is an automatic process and possibly influenced by unconscious memory [2]. 

The automaticity hypothesis [2], in regard to the role of attention allocation in encoding object-location memory, has been widely studied by comparing memory performance across *incidental* and *intentional* encoding conditions. In intentional encoding conditions, participants are explicitly instructed about a required subsequent retrieval phase, whereas in incidental encoding conditions, participants are shown an array of stimuli without awareness of a subsequent retrieval phase. Findings regarding memory performance are inconsistent; some studies have shown that the locations of objects are learned without participants receiving explicit instruction to remember the locations (e.g., [3]), thus supporting the automaticity hypothesis. Other studies have shown that intention to remember locations improves memory performance (e.g., [4]). Postma and colleagues [1] speculated that each component of object-location memory differs in its processing automaticity, with the spatial-location component operating more automatically than the object identity and object-location binding processing components. 

Other attention allocation tasks involve differentiating between *divided* versus *full* attention. In divided attention tasks, participants are required to respond to both target and distractor stimuli, whereas in full attention tasks, participants are required to direct their attention to the target stimulus only [5]. Studies investigating memory have typically demonstrated that in various memory tasks, divided attention during incidental or intentional encoding reduces performance (e.g., [5,6,7,8]). However, more recently, it has been suggested that under specific conditions, divided attention may facilitate memory performance. For example, Nussenbaum, Amso, and Markant [9] have shown that increasing the number of distractors in a divided attention condition did not impair memory for the target content. Furthermore, when distractors contained information that conflicted with the target content, increasing the number of distractors actually enhanced participants’ memory. 

The effects of attention during object-location memory encoding have been studied in the realm of visual working memory. Visual working memory is mainly characterized by its limited capacity, therefore the maintenance of attention to visual items is important in our daily behavior [10]. Previous studies investigated whether features and locations are represented as integrated objects in our visual working memory under various attention conditions. For example, Treisman and Zhang [11] demonstrated that attended objects are bound to their locations, however visual memory for binding is not disrupted when attention is directed to irrelevant stimuli. As opposed to working memory, long term memory requires different cognitive mechanisms at encoding, storage, and retrieval. The role of attention during object-location long-term memory encoding has been scarcely studied. To our knowledge, only two studies have examined the role of attention during object-location memory encoding under divided and full attention conditions. One study presented participants with an array of actual objects, and incorporated a verbal arithmetic task as a distraction in the divided attention condition [12]. The second study used a paper-and-pencil task and a tone discrimination task as an auditory distraction [13]. Both studies demonstrated that, with intentional encoding instruction, participants performed better in the full attention condition than the divided attention condition. However, under the incidental encoding conditions, this finding was replicated only in the study that used the actual array of objects, and not in the paper-and-pencil paradigm study. Furthermore, in the paper-and-pencil study women performed better in incidental than in intentional memory. Ecuyer-Dab and Robert [14] suggest that women might employ different strategies for incidental and intentional encoding. While under incidental encoding conditions, women spontaneously encode the surrounding elements, under intentional encoding with explicit instructions to memorize objects, women use an alternative strategy. In other words, females and males differ in the attentional and perceptual mechanisms that they employ in memorizing objects locations. Under incidental conditions, females unintentionally encode detailed features of their surroundings, and later are able to retrieve a precise representation of it. In contrast, under intentional conditions females employ a different strategy, such as verbal labeling of stimuli. For example, Lewin and colleagues [15] failed to demonstrate sex differences in location memory for uncommon objects and speculated that the absence of the familiar female superiority was due to females’ difficulty in assigning verbal labels to the objects. In accordance with previous studies examining the role of attention in various memory tasks, these findings suggest that the influence of attention allocation on memory performance is not uniform, but rather that it is affected by the nature of the distractors—including the modality and the relatedness of the distractor to the target—as well as the nature and the modality of the target. 

Studies investigating the neural correlates of object-location memory have demonstrated the involvement of the right hippocampus in spatial binding [1]. Only a few studies have examined the neural correlates of implicit and explicit spatial memory. Whereas some studies have shown the importance of the medial temporal lobes [16] and the striatum [17] for implicit spatial memory, others have demonstrated the importance of the diencephalic and frontal regions for explicit object-location memory [18]. 

The role of attention in memory performance has been also studied through a central component of executive function: *executive attention* or *attention control*. Attention control refers to attentional processes that support the ability to sustain information in the presence of internal or external distractions [16]. There are several attention control abilities, including, attention restraint, attention constraint [17], and sustained attention [16]. Sustained attention refers to attention control processes needed to preserve attention and task engagement over time (also referred to as vigilant attention; [18,19,20]). Studies that have addressed the relation between sustained attention, measured by the psychomotor vigilance task, and working memory capacity revealed that sustained attention was positively correlated with working memory capacity (e.g., [21]). Unsworth and Robison [16] recently proposed a cognitive-energetic model to explain the relation between sustained attention and various cognitive constructs, including memory. The underlying notion of the model is that intensity of attention varies within and between individuals. The intensity of attention is influenced by both intrinsic and extrinsic motivation levels, overall arousal levels, and intrinsic alertness. When attention intensity levels are high, task engagement is high and control levels are optimal. In four experiments examining the relation between sustained attention and working memory capacity, Unsworth and Robison [16] showed that this relationship is mediated by variation in intrinsic alertness—the ability to voluntarily control the intensity of attention on a continuous basis. 

In the search for the underlying cause of reduced sustained attention, a phenomenon called vigilance decrement, two theories have been suggested: the under-load theory and the over-load theory [22,23,24]. In the under-load theory, the decrement is deemed to be due to boredom, mindlessness, or goal habituation [25], whereas in the over-load theory, vigilance decrement is considered to be due to mental fatigue and resource depletion. In order to examine these two theories, a few studies sought to investigate the influence of various working memory demands on vigilance decrement. For example, in Helton and Russell’s [26] study, participants performed a vigilance task while simultaneously performing either a verbal or spatial working memory task. The researchers found that the concurrent verbal and spatial working memory load impacted the vigilance decrement among the participants. They concluded that vigilance decrement was caused by high cognitive resource demands, thus supporting the over-load theory.

The role of executive attention has also been examined in relation to incidental and intentional memory. Kontaxopoulou and colleagues [27] assessed participants’ episodic memory performance via virtual reality stimuli, both incidentally and intentionally, using both verbal and visuospatial tests. Additionally, participants completed a neuropsychological battery assessing attention and executive functioning. The researchers found that almost all attentional and executive functioning measures were associated with participants’ incidental, but not intentional, memory performance. They further reported that aging affected incidental (but not intentional) encoding processes. Given these two findings, Kontaxopoulou and colleagues [27] proposed that the ability to effectively execute incidental memory processes is more strongly connected with the overall cognitive system than is the ability to carry out intentional memory processes, as indicated by the association found with attention and executive functions. Indeed, memory studies among aging populations have shown that low scores on memory tasks were correlated with reduced activation in the frontal lobes (e.g., [28]). Furthermore, imaging studies have shown that there is a positive correlation between executive functioning and prefrontal cortex volume (for a meta-analysis, [29]). Therefore, it is suggested that incidental encoding processes, which hold a more prominent function in our daily lives, are perhaps more influenced by executive attention than intentional encoding processes 

The role of attentional resources in memory performance has been previously investigated, especially among aging populations. The search for the source of memory decline in some memory functions, but not in others, led researchers to examine several hypotheses to explain the underlying mechanisms in memory decline [30]. One of the hypotheses concerns the role of reduction in attentional resources in episodic memory (e.g., [30,31]), and the emphasis on stability of attention during encoding of items in recall and recognition tasks [32]. Previous studies demonstrated that aging is associated with long-term episodic memory decline, including object-location memory, and is characterized by reduction in attentional resources in episodic memory. Therefore, the present study aims at fine-tuning the conditions under which attentional resources influence memory performance among young adults, in order to uncover the processing mechanisms needed for executing essential functions in our daily lives, such as object-location memory. Specifically, the present study aimed to explore the role that executive attention, especially sustained attention, plays in incidental and intentional object-location memory performance. In line with the over-load theory, the present study was conducted under conditions of divided attention. Increasing mental demand among the participants enabled us to pinpoint the exact conditions under which vigilance decrement influenced performance on incidental and intentional memory encoding tasks. To the best of our knowledge, this study is the first to examine the association between these variables. Whereas a previous study [27] investigated the role of executive attention in relation to incidental and intentional memory (verbal and visuospatial), the present study is the first to examine this relation with the outcome of object-location memory, and under conditions of cognitive load. 

Furthermore, previous studies have demonstrated that getting a sufficient amount of sleep per night, on a regular basis, has an important influence on behavioral alertness and cognitive performance [33]. Therefore, in the current study, the quality and quantity of sleep during the four nights prior to the experiment were measured and used as an indicator of sleep deprivation and fatigue on the morning of the experiment. 

In summary, previous studies did not investigate object-location memory performance under cognitive load. Furthermore, to the best of our knowledge only one study assessed the role of attentional and executive functioning in incidental and intentional episodic memory [27]. However, it is not yet known whether sustained attention influences incidental and intentional episodic memory under cognitive load. Therefore, in the present study, including cognitive load allowed for an examination of whether sustained attention influences memory performance. Furthermore, the present study allowed us to examine whether sustained attention influences incidental as well as intentional encoding. Moreover, the present study controlled for the quality and quantity of sleep that have been related to cognitive performance. 

Our main hypotheses are:

(1) Memory performance would be higher under incidental encoding as compared to intentional encoding.
(2) Sustained attention, as measured by the psychomotor vigilance test, would be associated with incidental, but not intentional, encoding measures.

## 2. Method

### 2.1. Participants

Forty-nine female students (mean age 24.5 ± 1.89) from a college in the north of Israel participated in the study. Participants were recruited through advertisements at the college, and received course credit for their participation. We chose to recruit a female sample given the extensive body of research suggesting females’ superior performance in object-location memory tasks as compared to males (for a meta-analysis, see [34]). 

### 2.2. Materials 

The study was approved by the institutional review boards (IRBs) of the college (no: EMEK YVC2018-20 ). All participants arrived at the lab between 8:00 a.m. and 12:00 p.m., after four nights with ActiGraph, to participate in the experiment. After providing informed consent, participants completed a brief demographic questionnaire and a number of tasks. The tasks are outlined below.

*Object location memory–Incidental encoding*: The study included a stimulus array of 25 black-and-white drawings of objects, based on those used in the Eals and Silverman [35] study. The stimuli were presented on standard-size A4 white paper. Participants were instructed to complete both a pricing task and a distraction task within a one-minute time-frame. In the pricing task, which was designed to manipulate incidental (non-directed) encoding, participants were asked to write a price tag for each object directly on the paper. They were told that if they were unable to estimate a price for the object, they should provide a guess [36]. In the distraction task, designed to increase mental load, a pre-recorded soundtrack of piano tones, randomized by pitch (low or high), was presented in intervals of 2 or 3 s [37]. The participants were asked to indicate the low-pitched tone by raising their left hand and the high-pitched tone by raising their right hand. Immediately afterward, the participants were shown another stimulus array, in which 14 of the objects were in different locations than before. They were given 60 s to mark which objects’ locations were unchanged and circle the ones whose positions had changed. A manipulation check indicated that the participants were not suspicious about the purpose of the experiment. 

*Object location memory–Intentional encoding*: The design of the intentional encoding condition, including all of the materials presented, was identical to the incidental encoding task phase, with a new stimulus array. However, the one difference was that in the intentional encoding task, participants were given one minute to “try to memorize as many objects in the array as possible and their approximate locations” ([35], p. 100) before the distraction task was introduced. 

In the present study, the paper-and-pencil format was chosen due to task requirements of the incidental encoding condition (participants were asked to write a price estimation per each item). Given the limited timeframe (60 s) and the potential variation in computer skills across participants, a paper-and-pencil format (previously used for the same study purposes, e.g., [37]) was utilized. 

### 2.3. Psychomotor Vigilance Test (PVT): 

Participants completed a visual psychomotor vigilance task (PVT). This task is sensitive to sleep loss and circadian phase [38,39]. The psychomotor vigilance task (PVT) has been employed for the last 30 years as a sensitive test of sustained attention [40]. This simple measure of reaction time (RT) to repetitive stimuli has become recognized as a highly sensitive effective tool for measuring degradation of sustained attention performance under sleep deprivation or change in circadian phase [38,39]. The PVT is the most widely used measure of behavioral alertness. The standard duration of the PVT is 10 min; however, the optimal duration of the PVT is shorter than 10 min. Most studies use PVT outcomes to monitor sensitivity to total and partial sleep loss [41,42], and to differentiate sleep-deprived subjects from alert subjects [43]. 

However, in the present study the use of the PVT was for a different purpose (besides evaluating sensitivity to sleep loss; [22]); the PVT was used to explore the role that sustained attention plays in incidental and intentional object location memory performance. The PVT-B is a validated measure of sustained attention, with high test–retest reliability and low learning effects [19]. Previous studies validated the PVT as sensitive in differentiating dementia patients from healthy controls, and has been used among aging and MCI populations [44,45].

The PVT-B (Joggle Research Program, Seattle, WA, USA) is a sustained attention reaction time task, and it was performed on an iPad in the current study. Participants were instructed to maintain *vigilant attention* on a target box and respond as quickly as possible to the appearance of a stimulus, while avoiding responding prematurely. The outcome measure of the current study was the *Aggregate Score.* A score metric that penalized participants based on the percentage of responses that were lapses and the percentage of responses that were considered to be early response errors was calculated. The calculation was as follows: Aggregate Score = (1 − (Lapses/Responses) − (Errors/Responses)) × 100.

With regard to executive attention, it is important to emphasize that there is extensive evidence that the neurobehavioral consequences of sleep loss can be measured through certain aspects of cognitive functioning [46,47,48]. Among the most reliable effects of sleep deprivation is degradation of attention [38,49], including vigilant attention [38,50].

For each trial, an empty box was presented on an iPad screen, triggering a millisecond counter. Participants were requested to press on the screen to stop the counter. Participants were instructed to respond as quickly as possible, but to avoid pressing on the screen when the counter was not displayed (i.e., false starts). The inter-stimulus interval, defined as the period between the last response and the appearance of the next stimulus, varied randomly from 2–10 s [49].

*Sleep patterns*–The purpose of the objective sleep test was to control the quality and quantity of sleep, and to ensure that subjects did not suffer from sleep deprivation during the four days prior to the study.

Objective sleep patterns were measured using an actigraph (AMI, NY). This small device measures sleep patterns in one’s natural environment and provides objective data of one’s sleep patterns. Participants wore the actigraph in the four nights preceding the experiment. Actigraph recordings provided an estimation of participants’ sleep onset, wake time, sleep latency, sleep duration, true sleep minutes, wake after sleep onset (WASO), and sleep efficiency. 

### 2.4. Procedure

Prior to arriving to the lab for the experiment, participants’ objective sleep patterns were measured for four consecutive nights. Participants were then individually invited to the lab. All participants completed the study in the same room, The experiment utilized a crossover design, such that half of the participants performed the PVT-B task first, followed by the memory tasks, and the other half of the participants performed the tasks in the opposite order. Due to the study design (which included an incidental encoding condition), all participants performed the memory tasks (under incidental and intentional conditions) in the same order.

### 2.5. Power Analyses

G Power 3 (Heinrich Heine University, Düsseldorf, Germany) was used to determine the sample size required to find a significant difference in object-location memory performance between incidental and intentional encoding. The power analysis indicated that 47 participants would be needed to detect a medium effect size (dz = 0.5) with an alpha level of 0.05 and 90% power.

## 3. Results

### 3.1. Sleep Measures

Participants’ objective sleep patterns were characterized by sleep measures that fell within the typical ranges, a sleep duration that matched the recommended duration, and a high quality of sleep (i.e., sleep efficiency was high; see Table 1).

### 3.2. Incidental vs. Intentional Memory Performance

**Figure 1:**
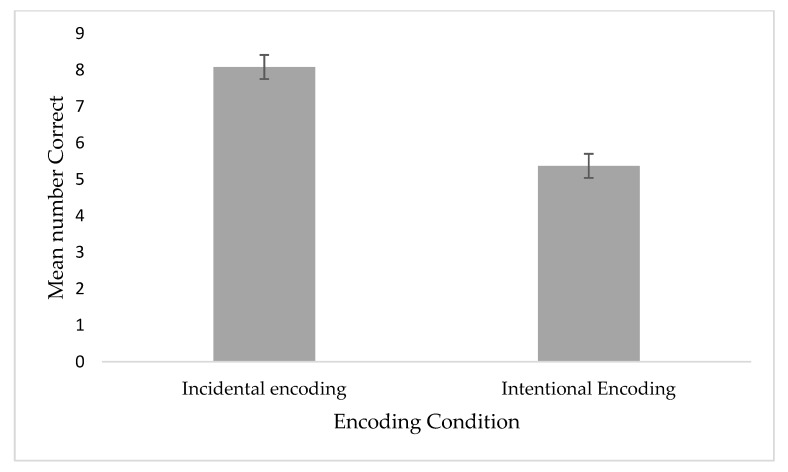
Mean number of correctly detected location-exchanged objects under divided attention incidental and intentional encoding conditions for the total scores. Error bars represent standard errors of the mean (SEM).

**Figure 2:**
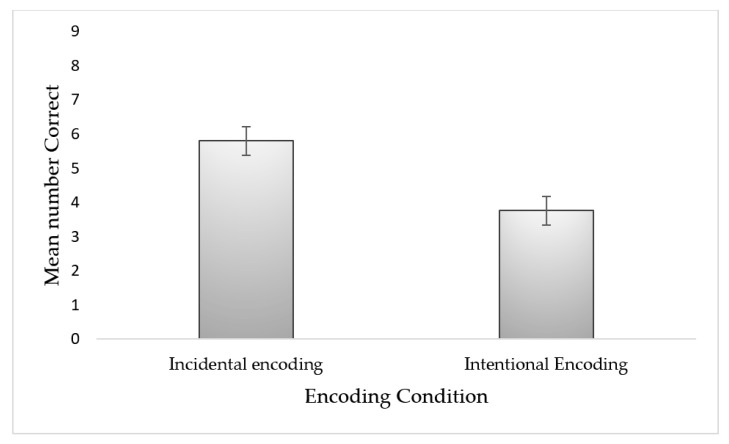
Mean number of correctly detected location-exchanged objects under divided attention incidental and intentional encoding conditions for the corrected scores. Error bars represent standard errors of the mean (SEM).

All the analyses were based on non-parametric statistics since location memory scores (incidental and intentional) were not normally distributed. Object-location memory performance across the incidental and intentional encoding conditions were calculated as two scores. The first was the total number of correct identifications of exchanged objects, as customarily used in the literature (e.g., [35,51]). The second was calculated as the number of correct identifications minus the number of incorrect identifications [52]. To test the difference in object-location memory performance across the incidental and intentional encoding conditions, we performed a Wilcoxon signed-rank test and found a significant effect, both for the total score (Z = 4.72, *p <* 0.00*1, effect size* = 0.67) (see Figure 1), and for the corrected score (Z = 3.82, *p <* 0.00*1, effect size* = 0.61) (see Figure 2). Participants scored higher on location memory in the incidental encoding condition as compared with the intentional encoding condition.

### 3.3. PVT-B Measures

Table 2 displays the mean and standard deviations of PVT-B measures.

### 3.4. The Role of Sustained Attention in Incidental vs. Intentional Memory Performance

**Figure 3:**
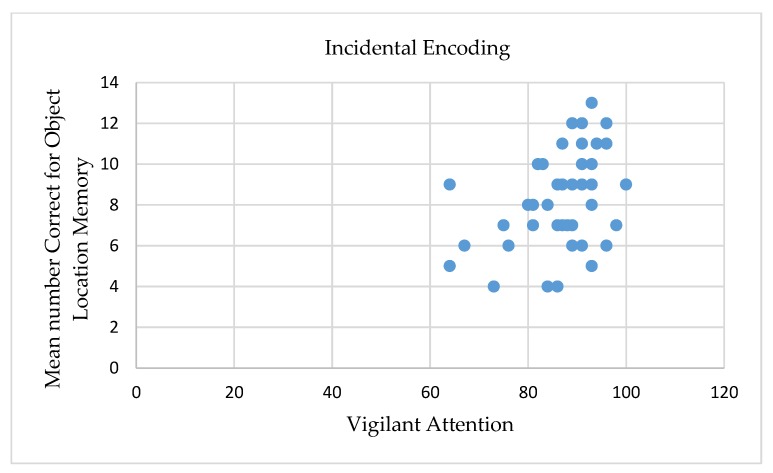
Mean number of correctly detected location-exchanged objects under the divided attention incidental encoding condition for the total scores as a function of vigilant attention (aggregate score).

**Figure 4:**
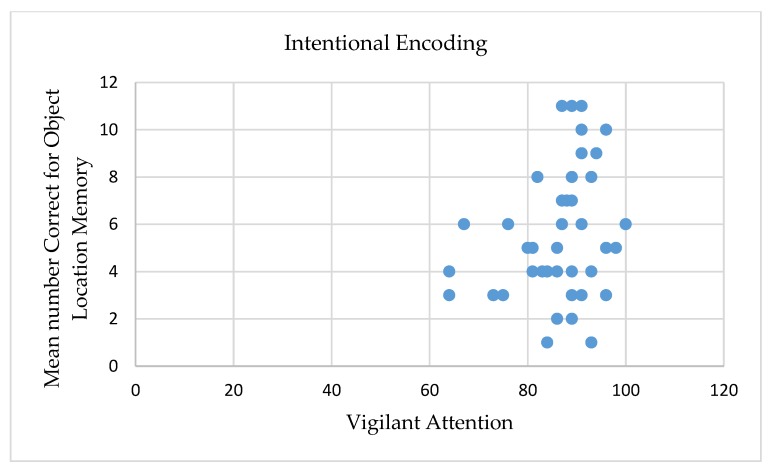
Mean number of correctly detected location-exchanged objects under the divided attention intentional encoding condition for the total scores as a function of vigilant attention (aggregate score).

First, correlation was performed between incidental and intentional memory performance. No significant correlation was found (*r* = 0.27, *p* > 0.05). Next, correlations were performed between sustained attention, as measured by the psychomotor vigilance test, and incidental and intentional memory performance for the total score and for the corrected score. Significant correlations were found only in the incidental encoding condition. Positive correlations were found between the aggregate score on vigilant attention and memory performance, with higher scores on vigilant attention correlating with higher scores on both scores of object-location memory. None of the correlations between vigilant attention and memory performance in the intentional encoding condition were significant (see Table 3). The difference between these correlations was not statistically significant (*p* > 0.05; see Figure 3 and Figure 4). 

## 4. Discussion

The present study aimed to examine performance differences in incidental and intentional memory under divided attention conditions. Furthermore, the present study sought to examine the relation between sustained attention and incidental and intentional memory performance. With regard to memory performance under conditions of incidental and intentional encoding, spatial memory studies are characterized by long-lasting controversies. Whereas automaticity hypothesis supporters suggest that the encoding of objects’ locations can occur even without attention allocation [2], other studies find that participants’ awareness of subsequent retrieval requests can improve performance (e.g., [4]). The present study provides support for the automaticity hypothesis showing that participants performed better in incidental than in intentional memory. Although previous studies that have shown that intention to learn locations improves memory performance, participants exhibited the ability to encode locations without explicit instruction to do so. The present study was conducted using a within-subject design, wherein participants responded both to the incidental condition and the intentional condition. A previous study [13] using the same paper-and-pencil format but with between-subject design, found the same results: female subjects under the incidental condition performed better than those under the intentional condition. Moreover, in the present study, participants memorized objects’ locations under attention load, during which they were additionally requested to direct their attention to an auditory task. Even though their attention resources were limited due to the need to allocate attention to another task, the distraction task did not deplete their attention resources, as evidenced by their ability to encode object locations to memory in incidental conditions. 

The present study additionally focuses on the relation between sustained attention and incidental and intentional memory performance. We found that sustained attention measures correlated with incidental, but not intentional, object-location memory performance. Our findings are in line with a recent study that examined the role of executive attention in relation to incidental and intentional memory, on both verbal and spatial tasks [27]. 

Furthermore, the present study examined memory performance under attention load conditions through a divided attention paradigm in order to uncover the role of sustained attention on wide conditions of memory encoding. Sustained attention is one of several attentional control, or executive attention, abilities [16]. Brain imaging studies implicate that several regions are associated with sustained attention, especially the anterior cingulate cortex and the right prefrontal cortex [53,54]. Additionally, Smith and colleagues [55] reported greater activity in the right hemisphere during tasks of spatial working memory, thus suggesting a coupling between memory demands and sustained attention [56]. However, the association between sustained attention and memory performance was found in the present study for the incidental encoding condition only. Based on Kontaxopoulou and colleagues’ [27] finding that aging affects incidental, rather than intentional, encoding processes, they proposed that the ability to effectively execute incidental memory processes is more strongly connected with the overall cognitive system, as indicated by the association found between incidental memory and attention and executive functions. Indeed, memory studies conducted with elderly populations have shown that low scores on memory tasks were associated with reduced activation in the frontal lobes (e.g., [28]). Furthermore, imaging studies support a positive correlation between executive functioning and prefrontal cortex volume (for a meta-analysis, see [29]). Further support comes from animal studies. For example, Parnell, Grasby, and Talk [57] demonstrated that lesions on the medial prefrontal cortex impacted incidental encoding for locations in rodents. The authors suggested that the prefrontal cortex is needed for sustained attention to incidental encoding of locations. 

In the realm of spatial attention, a paradigm termed *contextual cueing* [58] has been widely studied. In implicit contextual cueing tasks, repeated visual context facilitates visual search for target objects. Extensive research aimed at investigating the factors influencing spatial attention has been conducted. Researchers found that current goals, perceptual salience, statistical learning, reward, motivation and emotion affect attention [59]. Recent studies that have examined the influence of memory load on contextual cueing have presented conflicting findings. Whereas some studies have shown that contextual cueing is impaired by memory load [60], other work has shown that contextual cueing remains intact [61], suggesting that contextual cueing is sensitive to memory load under specific conditions [59]. 

The present study has some limitations. First, the present study focused on female participants only. Given previous results indicating sex differences in object-location memory performance and specifically the female advantage in these tasks, we chose to focus on females. However, future studies should expand the sampling frame to include male participants; this will allow for uncovering sex differences in processing strategies utilized in memory encoding and sustained attention. Second, the present study focused on divided attention conditions. The object-location memory literature has typically explored encoding manipulations under full attention conditions. Only scarcely have divided attention conditions been used [12,13]. In order to shed light on the role of sustained attention in memory encoding, the present study chose to utilize an attention load paradigm using divided attention conditions. However, to deepen our understanding on the role of attention control on memory encoding processes, a broader examination including various attention conditions (e.g., full, selective) is still needed. Third, the present study used a distraction task which did not appear to dilute the attentional resources allocated for the memory task, as illustrated by the relatively high performance of the participants. Future studies should examine various distraction modalities, including various component numbers in order to identify the precise conditions under which attentional resource allocation facilitates, as opposed to inhibits, memory performance. Fourth, although this has been customary in previous studies, the use of only one measure in the present study raises the need for replication in future studies using two measures forming sustained attention score. Fifth, due to study requirements (in the sake of face validity), all participants performed the study tasks (under incidental and intentional conditions) in the same order. Although the current findings replicate previous results found using between-subject design [13], confounding factors cannot fully be ruled out. Therefore, caution should be exercised interpreting the present findings.

## 5. Conclusions

In sum, the present findings demonstrated that in incidental encoding conditions, the distraction task did not completely diminish participants’ attentional resources, as they exhibited high memory performance. Furthermore, the present findings suggest that memory encoding also benefits from explicit instructions to memorize locations under divided attention conditions, but to a lesser extent. Therefore, the present study supports the notion that object-location memory possesses several components that differ in processing automaticity. Furthermore, to the best of our knowledge, the present study is the first to examine the relation between sustained attention and object-location memory. We found that sustained attention plays an important role in incidental, but not in intentional, encoding, thus supporting previous findings which have examined other memory tasks. In addition, in line with the over-load theory, the present study was conducted under conditions of increased mental load. We found that under conditions of divided attention, executive attention was associated with incidental, but not with intentional encoding. These findings enable us to identify the exact conditions under which executive attention influences memory performance. Studying object-location memory through specifying the conditions by which performance declines is essential regarding aging influences on memory performance. Moreover, previous findings in animal and human imagery studies have shown that executive attention and incidental memory performance are connected with the same brain regions, including the prefrontal cortex in particular. Future studies should focus on other incidental memory tasks prominent in our daily lives and their relation to executive attention. A large body of research has demonstrated that object-location memory is even more susceptible to age-related declines than target memory [62,63]. Further examination of the role of executive attention on object-location memory, and the corresponding neural infrastructure, will shed light on the potential deleterious effects of aging on memory.

## Figures and Tables

**Table 1:** Actigraphic sleep pattern.

	Mean ± SD
**Sleep Onset**	00:22 ± 1.09
**Wake Time**	8:47 ± 0.89
**Sleep Latency**	13.92 ± 12.13
**Sleep Duration**	445.93 ± 65.74
**True Sleep Minutes**	410.35 ± 61.97
**Waso (min)**	15.83 ± 13.41
**Sleep Efficiency (%)**	96.17 ± 3.0

**Table 2:** PVT-B measures.

	Mean ± SD
**Responses**	44.48 ± 2.21
**Errors**	2.37 ± 3.18
**Aggregate Score**	85.81 ± 13.77

**Table 3:** Correlations between vigilant attention (aggregate score) and location-exchanged objects during divided attention incidental and intentional encoding conditions.

	Incidental Encoding	Intentional Encoding
Total scores	0.30 *	0.12
Corrected scores	0.29 *	0.15

* *p* < 0.05; The total score was the total number of correct identifications in exchanged objects The corrected score was the total number of correct identifications minus the incorrect identifications in exchanged objects.
